# Comparative Evaluation of the Cytotoxicity of Glyphosate-Based Herbicides and Glycine in L929 and Caco2 Cells

**DOI:** 10.3389/fpubh.2021.643898

**Published:** 2021-05-07

**Authors:** Francesca Truzzi, Daniele Mandrioli, Federica Gnudi, Paul T. J. Scheepers, Ellen K. Silbergeld, Fiorella Belpoggi, Giovanni Dinelli

**Affiliations:** ^1^Department of Agricultural Sciences, University of Bologna, Bologna, Italy; ^2^Cesare Maltoni Cancer Research Center (CMCRC), Ramazzini Institute (RI), Bologna, Italy; ^3^Radboud Institute for Health Sciences, Radboud University Medical Center (UMC), Nijmegen, Netherlands; ^4^Bloomberg School of Public Health, Johns Hopkins University, Baltimore, MD, United States

**Keywords:** mechanism, glyphosate, cancer, toxicity, biomarker

## Abstract

**Introduction:** Glyphosate, an amino acid analog of glycine, is the most widely applied organophosphate pesticide worldwide and it is an active ingredient of all glyphosate-based herbicides (GBHs), including the formulation “Roundup. ” While glycine is an essential amino acid generally recognized safe, both epidemiological and toxicological *in vivo* and *in vitro* studies available in literature report conflicting findings on the toxicity of GBHs. In our earlier *in vivo* studies in Sprague–Dawley rats we observed that exposure to GBHs at doses of glyphosate of 1.75 mg/kg bw/day, induced different toxic effects relating to sexual development, endocrine system, and the alteration of the intestinal microbiome. In the present work, we aimed to comparatively test in *in vitro* models the cytotoxicity of glycine and GBHs.

**Methods:** We tested the cytotoxic effects of glycine, glyphosate, and its formulation Roundup Bioflow at different doses using MTT and Trypan Blue assays in human Caco2 and murine L929 cell lines.

**Results:** Statistically significant dose-related cytotoxic effects were observed in MTT and Trypan Blue assays in murine (L929) and human (Caco2) cells treated with glyphosate or Roundup Bioflow. No cytotoxic effects were observed for glycine. In L929, Roundup Bioflow treatment showed a mean IC50 value that was significantly lower than glyphosate in both MTT and Trypan Blue assays. In Caco2, Roundup Bioflow treatment showed a mean IC50 value that was significantly lower than glyphosate in the MTT assays, while a comparable IC50 was observed for glyphosate and Roundup Bioflow in Trypan Blue assays. IC50 for glycine could not be estimated because of the lack of cytotoxic effects of the substance.

**Conclusion:** Glyphosate and its formulation Roundup Bioflow, but not glycine, caused dose-related cytotoxic effects in *in vitro* human and murine models (Caco2 and L929). Our results showed that glycine and its analog glyphosate presented different cytotoxicity profiles. Glyphosate and Roundup Bioflow demonstrate cytotoxicity similar to other organophosphate pesticides (malathion, diazinon, and chlorpyriphos).

## Introduction

Glyphosate [IUPAC chemical name N-(phosphonomethyl)-glycine], an amino acid analog of glycine, is the most widely applied organophosphate pesticide worldwide and it is an active ingredient of all glyphosate-based herbicides (GBHs), including in the formulation “Roundup” (1, 2). It is mainly marketed as a broad-spectrum systemic herbicide and crop desiccant (3). Glyphosate was in fact synthesized in 1950 by a Swiss chemist, Henri Martin, as an analog of the non-essential amino acid glycine, but its herbicidal properties were not discovered for another 20 years (4). The massive and increasing use of GBHs leads to a global burden of occupational exposures in manufacturing workers and GBH applicators (farmers), as well as increasing exposures in the general population, as demonstrated by environmental contamination from glyphosate residues found in air (5), groundwater (6, 7), drinking-water (8), crops (9, 10), food (11, 12), and animal feed (13). In humans, the main exposure routes to glyphosate are inhalation and dermal exposure in the occupational setting and for the general population consumption of contaminated drinking water and residues in food items (14).

The results of oral studies with [^14^C] glyphosate in rats, rabbits and goats indicate that absorption from the gastrointestinal tract is incomplete and amounts to up to 30% of the dose (15–17). The most relevant routes of excretion following oral administration of glyphosate [^14^C] are feces (70–80%) and urine (20–30%) (18). Therefore, most of the glyphosate assumed orally is not absorbed in the gastro-intestinal tract and is then excreted with the feces. On the other hand, glycine is very rapidly absorbed along the gastrointestinal tract via special carrier systems and then transported via the portal vein into the liver but also distributed within the whole body since it is involved in the body's production of haem, DNA, phospholipids, and collagen (19).

In March 2015, the World Health Organization's International Agency for Research on Cancer (IARC) classified three organophosphates (glyphosate, malathion, and diazinon) as “probably carcinogenic for humans” (Category 2A) (20). In contrast, in November 2015 the European Food Safety Agency determined glyphosate was “unlikely to pose a cancer risk for man” (EFSA 2015). In 2018 the European Chemicals Agency (21) Risk Assessment Committee concluded that “the scientific evidence so far available does not satisfy the criteria for classifying glyphosate as carcinogenic, mutagenic or toxic for reproduction” (21). In 2019 a US federal health agency, the Agency for Toxic Substances and Disease Registry (ATSDR) (22), part of the Centers for Disease Control and Prevention (23), determined that both cancer and non-cancer hazards derive from exposure to glyphosate and GBHs. The ATSDR 2019 report clearly lays out the vast array of scientific evidence linking both pure glyphosate (rodent studies) as well as formulations (in human epidemiologic studies) to cancer. In fact glyphosate, as the pure active substance, and its formulations may not have the same toxicity. Glyphosate formulations contain a number of so-called “inert” ingredients or adjuvants to facilitate the uptake by plants, most of which are patented and not publicly known (in many countries the law does not require a full disclosure of pesticide ingredients). GBHs that contain surfactants and adjuvants might act differently than glyphosate alone (24–26). In fact, adjuvants might be toxic in their own right and potentiate the toxic effects of glyphosate, as in the case of polyethoxylated tallow amine (POEA), that have been banned in the EU since 2016 (27–31).

While glycine is an essential amino acid generally recognized safe, both epidemiological and toxicological *in vivo* and *in vitro* studies available in literature report conflicting findings on the toxicity of GBHs. In our previous *in vivo* studies on Sprague–Dawley rats we observed that exposure to GBHs (pure glyphosate and Roundup Bioflow) at doses of glyphosate considered to be “safe,” the US ADI of 1.75 mg/kg bw/day, defined as the chronic Reference Dose (cRfD) determined by the US EPA, induced different toxic effects relating to sexual development, endocrine system, and the alteration of the intestinal microbiome (32–34). Furthermore, mechanistic data are increasingly important for hazard characterization, as exemplified by the 10 key characteristics of carcinogens considered by IARC in their evaluations, which include “Alters cell proliferation, cell death or nutrient supply” (35, 36). In particular, the MTT and Trypan Blue assays are routine and convenient methods for determination of cytotoxicity (37–39). MTT assay is a colorimetric assay of viable cells, while Trypan Blue assay is a dye exclusion staining assay. The combined and comparative use of MTT and Trypan Blue assays allows to overcome the limitations the single assays and increases the precision of the IC50 estimates derived from these studies (40–42). In the present work, we aimed to test the cytotoxic effects of glycine, glyphosate and its formulation Roundup Bioflow at different doses using MTT and Trypan Blue assays in human Caco2 and murine L929 cell lines.

## Materials and Methods

### Cell Cultures

L929 mouse fibroblasts (ATCC-CCL1) were cultured with Dulbecco's Modified Eagle Medium (DMEM, Gibco) added with 10%, fetal bovine serum (FBS, Gibco), 1 mM L-glutamine (Gibco), and 1% penicillin-streptomycin (Gibco). Caco2 human epithelial cell line (ATCC HTB-37) obtained from colorectal adenocarcinoma were cultured with DMEM with 10%, of FBS and 1% penicillin-streptomycin. Cells were grown in a monolayer condition at 37°C in an atmosphere of 5% CO_2_ and for the cell treatments, cells were cultured with DMEM alone.

### Test Substance

Glyphosate (Pestanal^TM^ analytical standard, CAS number 1071-83-6, purity > 99.5%) was obtained from Sigma-Aldrich (Milan, Italy). The commercial formulation Roundup Bioflow (containing 360 g/L of glyphosate acid in the form of 480 g/l isopropylamine salts of glyphosate (41.5%), water (42.5%), and surfactant (16%, ingredient not disclosed by the producer) was supplied from a local agricultural consortium (Consorzio Agrario dell'Emilia, Bologna, Italy). Glycine was supplied from Biosolve (Valkenswaard, Netherlands).

### Cell Treatments

Glycine (Biosolve), Glyphosate (Sigma-Aldrich), and Roundup^®^Bioflow were added to cells for 24 h. Glycine and Glyphosate were diluted in water and the treatment doses were prepared at different concentrations according the following formula:

$$\begin{array}{l} 175\;mg/kg\;bw\;(US\;NOAEL)\;\times \;70kg\;(average\;body\;weight\;\\ \;of\;an\;adult)/2\;Lt\;(daily\;water\;intake) \end{array}$$

The final concentration of the treatments was of 6.125 g/L [36.676 mmol/L]; 0.6125 g/L [3.667 mmol/L]; 0.06125 g/L [0.3667 mmol/L], and 0.006125 g/L [0.03667 mmol/L] (equivalent respectively to 175, 17.5, 1.75, and 0.175 mg/kg bw). Each dilution was prepared in DMEM and the pH of each final solution was corrected to 7.0 by adding NaHCO_3_ (Gibco).

### Measurement of Cell Proliferation for Adherent Cells (MTT)

Cell cytotoxicity was measured using the3-(4,5-dimetiltiazol-2-il)-2,5-difeniltetrazolio (MTT) assay. L929 cells (5 × 10^4^ cells/well) and Caco2 cells (10^5^ cells/well) were plated in 96-well tissue culture plate in complete medium (100 μL/well). The multiwell plates were incubated at 37°C, 5% CO_2_ for 24 h. After 24 h, the culture medium was removed and equal volumes (100 μL) of the treatments were added to each well. In control wells, 100 μL DMEM were added. Control wells consisted of untreated cell cultures. Twenty-four hours later, proliferative cells were detected by MTT assay, according to the ISO 10993-5 International Standard procedure (43). The main purpose of the ISO 10993-5 procedure is to define a scheme for testing *in vitro* cytotoxicity of different extracts according to a multi-step approach. Briefly, cells were incubated with MTT solution (1 mg/mL, Life Technologies) at 37°C for 2 h. Then, MTT solution was removed and cells were solubilized with 100 μl of isopropanol. The formazan dye formation was evaluated by scanning multiwell spectrophotometer at 540 nm. The results were expressed as percentage of viable cells compared to controls.

### Measurement of Cell Viability (Trypan Blue) and Average Cell Size

Cell viability was measured using the Trypan Blue assay. L929 and Caco2 cells were plated in 24-well tissue culture plate (50 × 10^5^ cells/well) in complete medium. The day after, treatments diluted in DMEM were added to cells for 24 h. To detect viability and cells size, cells were trypsinized with a solution of trypsin 0.05% and EDTA 0.02%. Cell were then carefully diluted in a 0.4% Trypan Blue (Gibco) solution by preparing a 1:1 dilution with the cell suspension. Viable cells were counted and average cell size analyzed by using Countess^®^II FL (Thermo Fisher Scientific). The results were expressed as percentage of viable cells compared to controls.

### Statistical Analysis

The MTT cell tests were carried with six replicates for each treatment and data were expressed as mean values of three different experiments. Statistical analysis was performed with R software (44). Normal and homoscedastic data were analyzed with ANOVA followed by pairwise comparison (Dunnett test) and Tukey *post-hoc* tests with Bonferroni correction. Non-normal homoscedastic data were analyzed with the non-parametric Kruskall–Wallis test and Dunn's *post-hoc* test with Bonferroni correction. Differences were considered to be significant at a *p* < 0.05. IC50 values were calculated by Probit regression.

## Results

### MTT: Effects of Glycine, Glyphosate, and Roundup Bioflow on L929 and Caco2 Cells Proliferation

**Figure 1:**
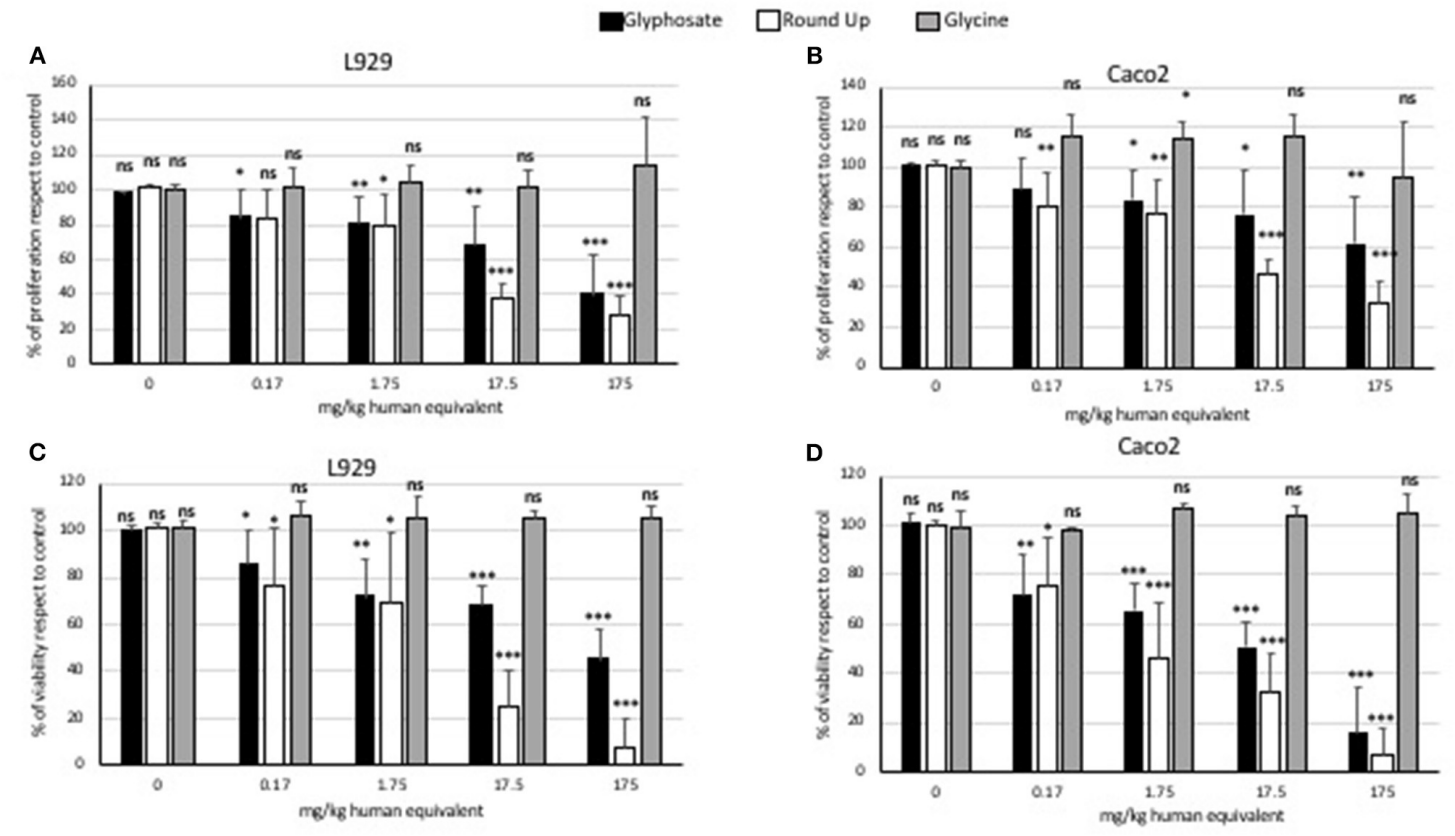
MTT (proliferation) assay and Trypan Blue (viability) assay: effects of pure glyphosate, Roundup Bioflow, and glycine at the dose range 0–175 mg kg^−1^ (human equivalent) on cell proliferation of L929 fibroblasts **(A)**, Caco2 cells **(B)**, and on cell viability of L929 fibroblasts **(C)** and Caco2 cells **(D)**. Data are expressed as mean value (± st. dev.) (% compared to control). Pairwise comparison based on Anova (Dunnett test). ns, not significant: *P < 0.05; **P < 0.01; ***P < 0.001.

**Figure 2:**
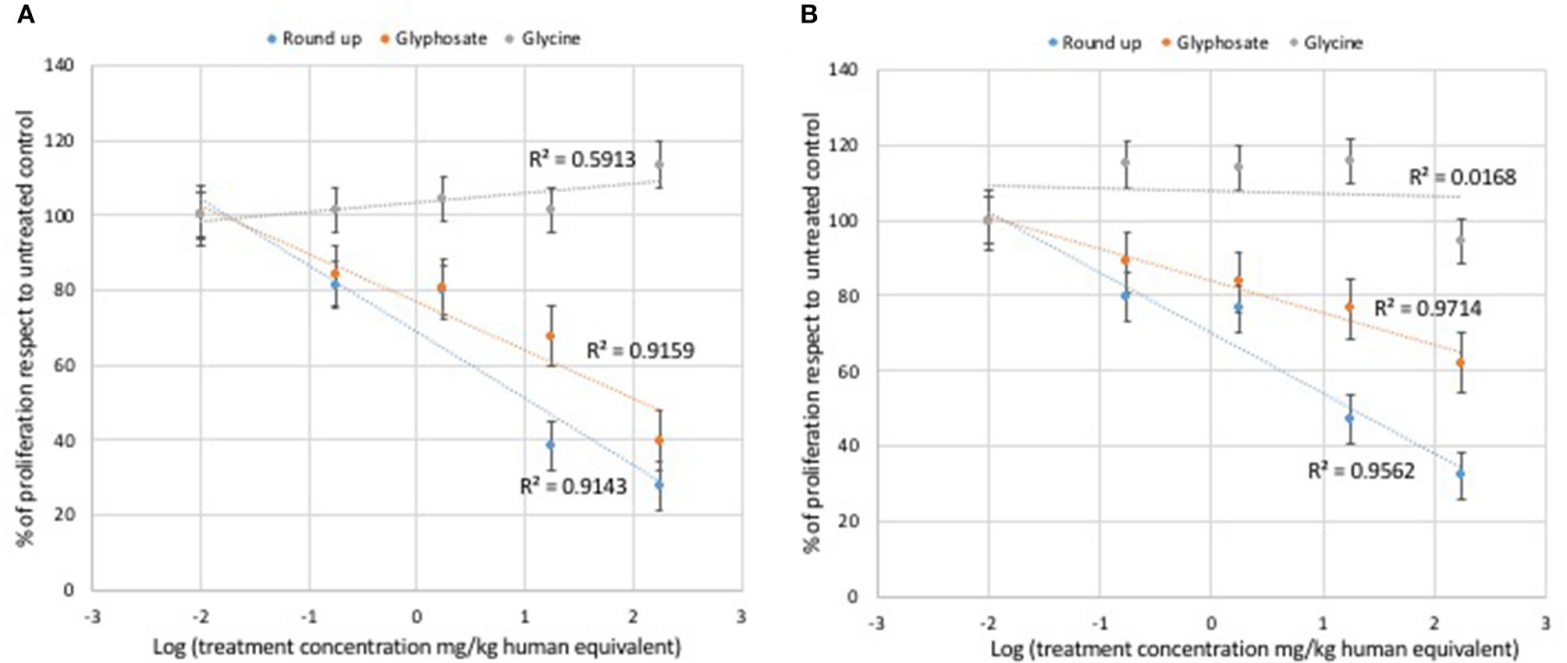
Relationship between pure glyphosate, Roundup Bioflow, and glycine at the dose range 0–175 mg kg^−1^ (human equivalent) (Log scale) and cell proliferation of L929 fibroblasts **(A)** and Caco2 cells **(B)**. Data are expressed as mean value (± st. dev.) (% compared to control). *R*^2^, coefficient of determination.

Glycine did not modulate either L929 or Caco2 cell proliferation at any of the concentrations used in the MTT assays (Figure 1). Statistically significant decreases of proliferating cells were observed at all doses of glyphosate compared to controls in both L929 models and Caco2 models, except at the lowest dose in Caco2 (Figure 1). Glyphosate showed also positive correlation between the percentage of proliferating cells as a function of the concentration for both L929 cells (*R* = 0.957, Figure 2A) and Caco2 cells (*R* = 0.986, Figure 2B). Statistically significant decreases of proliferating cells were observed at all doses of Roundup Bioflow compared to controls in both L929 models and Caco2 models, except at the lowest dose in L929 (Figure 1). Roundup Bioflow showed also positive correlation between the percentage of proliferating cells as a function of the pesticide concentration for both L929 and Caco2 cells, with *R* = 0.956 and 0.978, respectively (Figures 2A,B).

### Trypan Blue: Effects of Glycine, Glyphosate, and Roundup Bioflow on L929 and Caco2 Cells Viability

**Figure 3:**
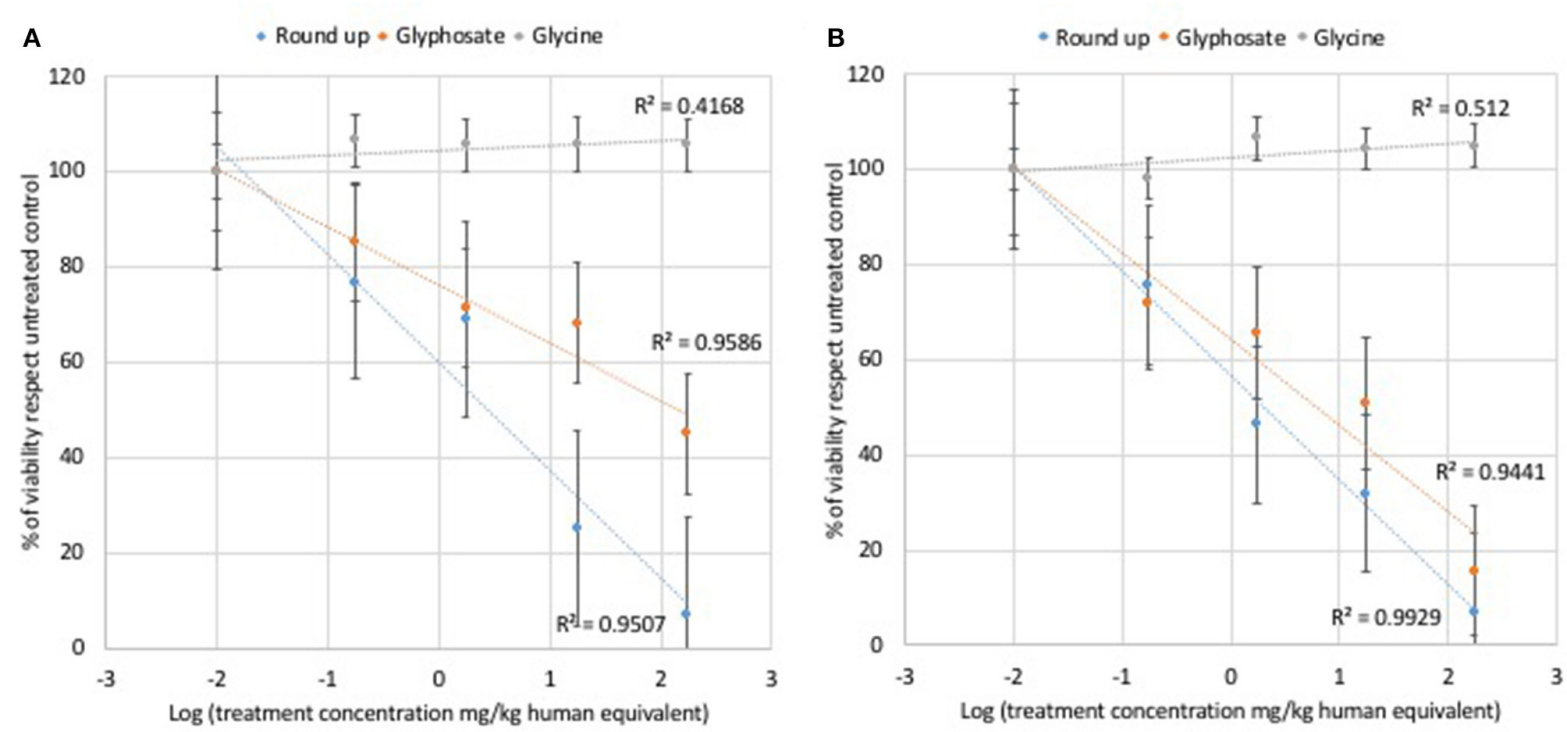
Relationship between pure glyphosate, Roundup Bioflow, and glycine at the dose range 0–175 mg kg^−1^ (human equivalent) (Log scale) and cell viability of L929 fibroblasts **(A)** and Caco2 cells **(B)**. Data are expressed as mean value (± st. dev.) (% compared to control). *R*^2^, coefficient of determination.

Glycine did not modulate either L929 or Caco2 cell viability at any of the concentrations used for cell treatments (Figure 1). Statistically significant decreases of vital cells were observed at all doses of glyphosate compared to controls in both L929 models and Caco2 models (Figure 1). Glyphosate showed also a positive correlation between the percentage of vital cells compared to control and the dose treatments both for L929 fibroblasts (*R* = 0.978, Figure 3A) and intestinal cell Caco2 (*R* = 0.972, Figure 3B). Statistically significant decreases of vital cells were observed at all doses of Roundup Bioflow compared to controls in both L929 models and Caco2 models (Figure 1). Roundup Bioflow showed also a positive correlation as a function of the pesticide concentration for both L929 and Caco2 cells with *R* = 0.975 (Figure 3A) and *R* = 0.996 (Figure 3B), respectively.

### Effects of Glyphosate and Roundup Bioflow on L929 and Caco2 Average Cell Size

**Figure 4:**
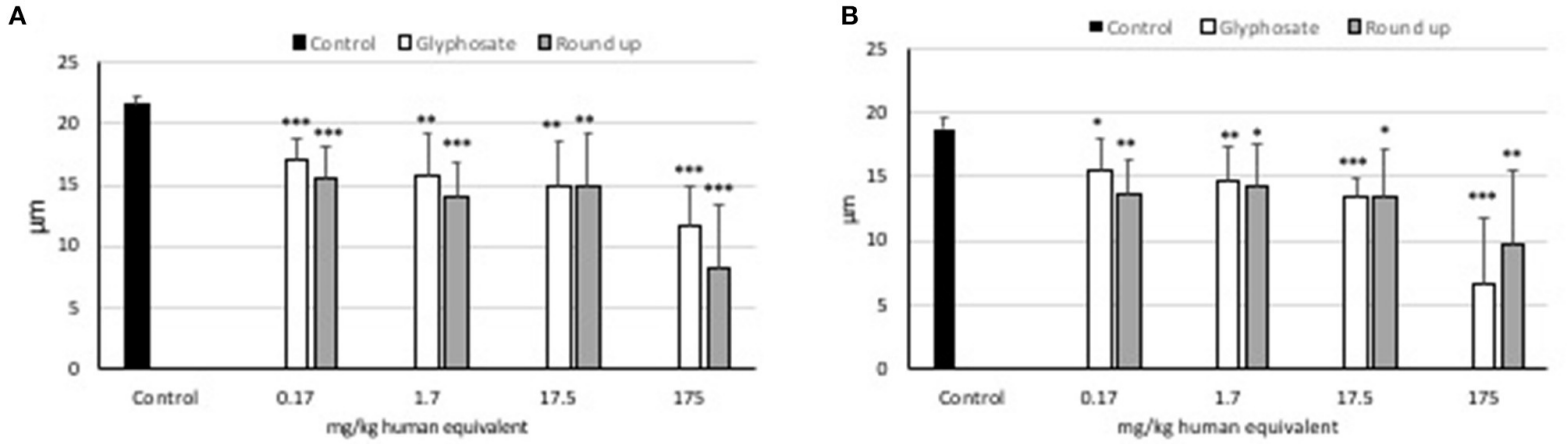
Effects of pure glyphosate and Roundup Bioflow at the dose range 0–175 mg kg^−1^ (human equivalent) on cell size of L929 fibroblasts **(A)** and Caco2 cells **(B)**. Data are expressed as mean value (± st. dev.). Pairwise comparison based on Anova (Dunnett test). ns, not significant: *P < 0.05; **P < 0.01; ***P < 0.001.

Statistically significant decreases of average cell size were observed at all doses of glyphosate compared to controls in both L929 models and Caco2 models (Figure 4). Statistically significant decreases of average cell size were observed at all doses of Roundup Bioflow compared to controls in both L929 models and Caco2 models (Figure 4).

### Correlation Between MTT and Trypan Blue Assays for L929 and Caco2 Cells Treated With Glyphosate and Roundup Bioflow

**Figure 5:**
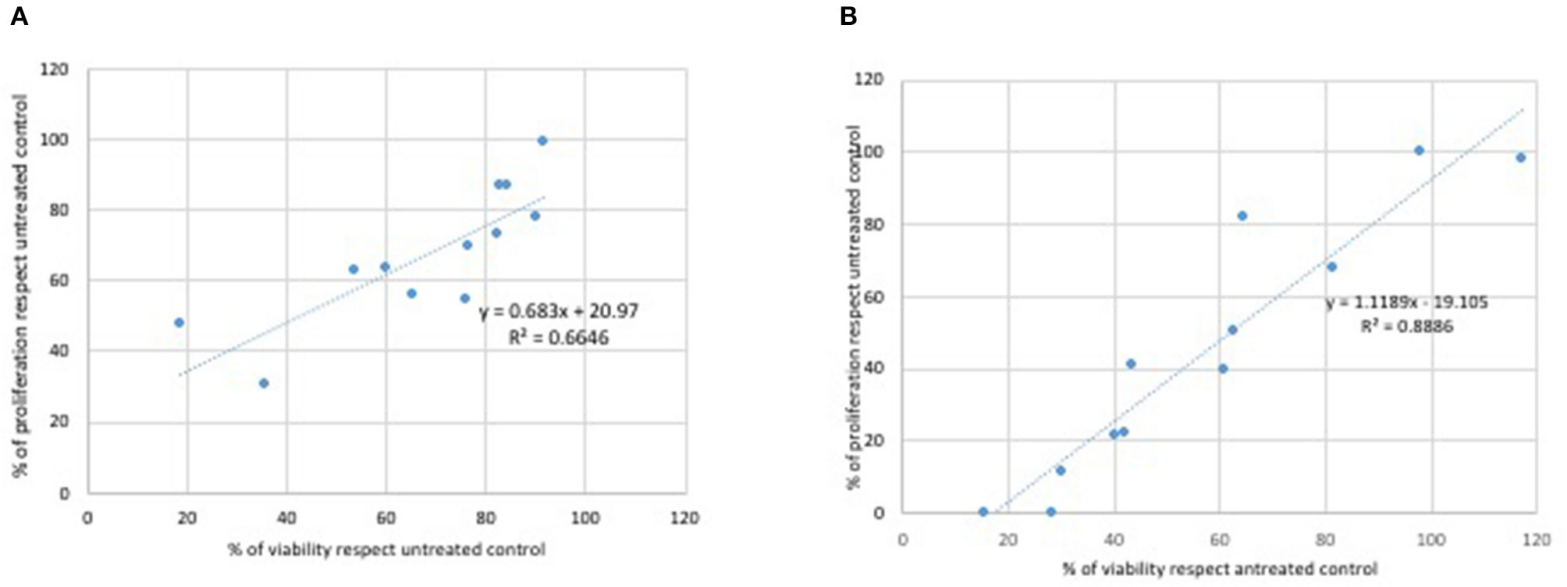
Correlation between MTT and Trypan Blue test for L929 cells treated with different doses of Glyphosate **(A)** and Roundup **(B)**. Both correlations were statistically significant at *P* < 0.05.

**Figure 6:**
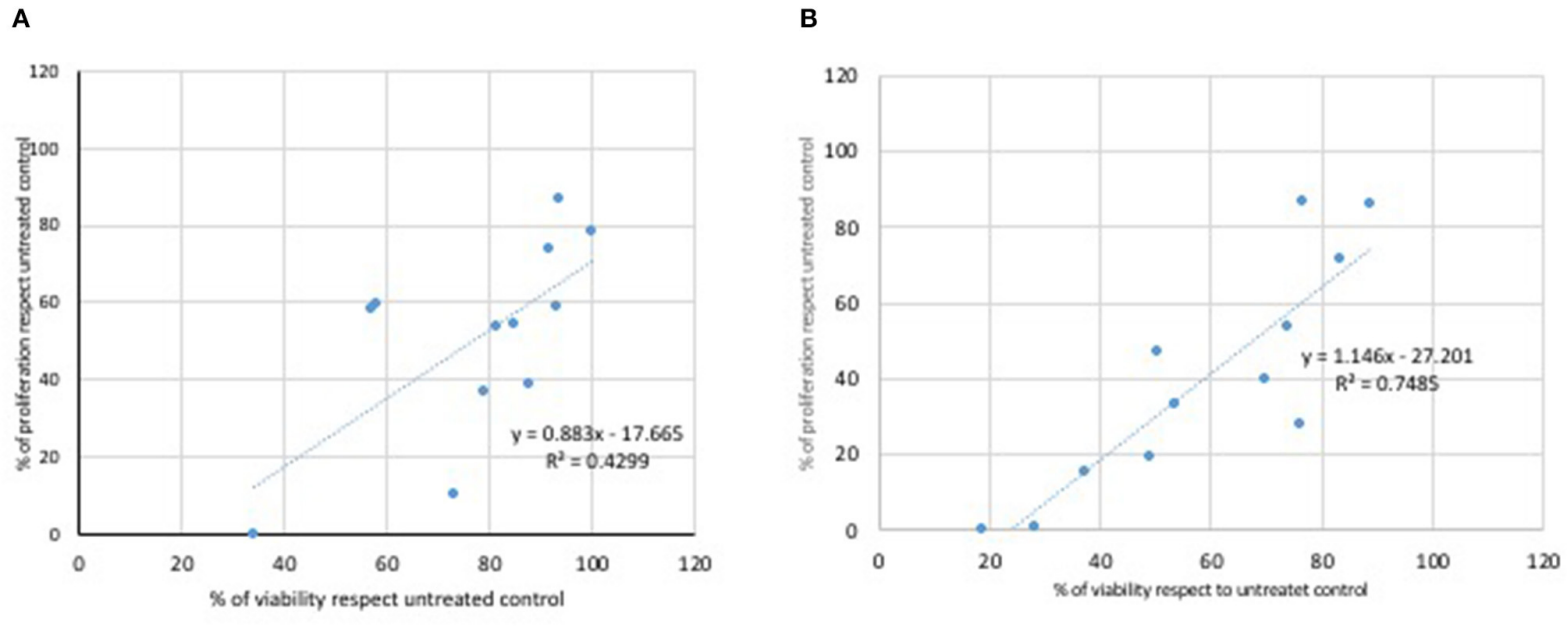
Correlation between MTT and Trypan Blue test for Caco2 cells treated with different doses of Glyphosate **(A)** and Roundup **(B)**. Both correlations were statistically significant at *P* < 0.05.

A correlation was performed between results obtained from the MTT assay and the viability analysis obtained by the treatment of L929 (Figure 5) and Caco2 cells (Figure 6) with Glyphosate and Roundup Bioflow. Regarding L929 fibroblasts, both Glyphosate (Figure 5A) and Roundup Bioflow (Figure 5B) treatments showed a positive correlation as a function of the concentrations, even if it resulted more evident when L929 fibroblasts were treated with Roundup Bioflow. Regarding Caco2 cell treatments, both Glyphosate (Figure 6A) and Roundup Bioflow (Figure 6B) treatments showed a positive correlation as a function of the concentrations even if it resulted more evident when L929 fibroblasts were treated with Roundup Bioflow. Analysis revealed that both of the correlations were statistically significant at *P* < 0.05.

### IC50 (ug/L) Mean Values Calculated for MTT and Trypan Blue on L929 and Caco2 Cell Lines

IC50 mean values for MTT and viability and size regarding the treatments of Glycine, Glyphosate, and Roundup Bioflow was calculated related L929 fibroblasts and Caco2 cells. For Glycine, it was not possible to calculate IC50 for either L929 fibroblasts or Caco2 cells. In L929 treatments (Table 1) Roundup Bioflow showed IC50 mean value significantly lower compared to the IC50 mean value induced by Glyphosate treatments for both the MTT and Trypan Blue assays. In Caco2 treatments (Table 2), Roundup Bioflow showed IC50 which was significantly lower than the one induced by Glyphosate for MTT, while statistical significance was not reached for differences relating viability assay.

**Table 1:** IC50 (μg/L) mean values calculated for MTT, viability on L929 cells.

	**MTT**	**Viability**
Glycine	ND	ND
Glyphosate	119.9 ± 38.5 (a)	147.9 ± 22.4 (a)
Roundup Bioflow	11.7 ± 5.3 (b)	2.8 ± 2.3 (b)

*One way Anova (different letters indicates statistically different mean values at P < 0.05)*.

**Table 2:** IC50 (μg/L) mean values calculated for MTT, viability on Caco2 cells.

	**MTT**	**Viability**
Glycine	ND	ND
Glyphosate	ND	6.2 ± 3.5 (a)
Roundup Bioflow	18.1 ± 6.3 (b)	1.9 ± 1.1 (a)

*One way Anova (different letters indicates statistically different mean values at P < 0.05)*.

## Discussion

The MTT and viability results confirm a different mechanism of action of glycine and its analog glyphosate (and its formulation Roundup Bioflow). In fact glycine did not show any sign of cytotoxicity, confirming the extremely safe profile of this substance, in line with the reported EU NOAEL (2,000 mg/Kg bw). On the other hand, glyphosate and its formulation Roundup showed a clear dose-related cytotoxic effects in MTT and viability assays in both Caco2 human intestinal cell line and L929 murine fibroblast cell line. The cytotoxic effects were also observed at doses that are lower than the current EU NOAEL (50 mg/Kg bw). Therefore, our results confirm previous evidence of cytotoxicity of glyphosate in *in vitro* models on Raji human hematological cell lines (45). In addition, our findings support the hypothesis of a higher toxic potency of the formulation, compared to pure glyphosate, in line with the results observed in other *in vitro* cytotoxicity studies by other authors (28, 46) and the results of our recent *in vivo* studies on Sprague–Dawley rats exposed to the same formulation (Roundup Bioflow) (34). Furthermore, different organophosphates other than glyphosate proved had similar effects in different *in vitro* models: in particular malathion exposure induced dose-dependent cytotoxic effects in MTT assays performed on HepG2 human liver cell line (47), diazinon exposure induced dose-dependent cytotoxic effects in MTT assays performed on HCT116 human intestinal cell line (48), chlorpyrifos induced dose-and time-dependent cytotoxic effects in MTT assays performed on SH-SY5Y human neural cell line (49). However, our experiment presents certain limitations: (1) direct quantitative extrapolations of IC50 values from cell lines to the human *in vivo* situation might under or overestimate the effects, therefore further studies are recommended to establish a dose-response relationship *in vivo* (50) (2) the equivalent dose was calculated based on the assumption that all of the substance administered would lead to exposure of the intestinal system (in the case of the intestinal model Caco2) or in a distant organ (the case of the fibroblast model L929), however the first is a probably more realistic exposure scenario for glyphosate in light of the relatively limited systemic absorption of glyphosate (and the high level of gastrointestinal exposure) (17), while the second might be a more realistic exposure for glycine that presents higher systemic absorption rates; (3) routes of exposures other than oral that are particularly relevant in the occupational setting, thus inhalation and/or skin absorption, were not taken into account in this model, although glyphosate can reach through this routes other organs and systems before being metabolized to AMPA; (4) we could not test separately the cytotoxicity of the adjuvants present in the formulation Roundup Bioflow as these are trade secrets.

## Conclusion

Glyphosate and its formulation Roundup Bioflow, but not glycine, caused dose-related cytotoxic effects in *in vitro* MTT and viability assays in human intestinal and murine fibroblast models (Caco2 and L929). Our results showed that glycine and its analog glyphosate presented different cytotoxicity profiles. Glyphosate and Roundup Bioflow demonstrate cytotoxicity similar to other organophosphates, in particular malathion, diazinon, and chlorpyriphos. Notably, glyphosate, diazinon, and malathion have been recently classified as probable carcinogen by IARC (Group 2A) (51). The formulation Roundup Bioflow seem to be more cytotoxic than pure glyphosate. Cytotoxic effects of GBHs were observed at doses that are lower than the current EU NOAEL (50 mg/Kg bw).

## Data Availability Statement

The original contributions presented in the study are included in the article, further inquiries can be directed to the corresponding author.

## Author Contributions

DM and FT prepared the first draft of manuscript. DM, FT, and GD designed the experiments. FT conducted the experiments. GD and FB supervised the work. GD performed statistical analyses and reviewed the manuscript. PS, ES, and FG critically revised the manuscript and originally contributed to the methods and the elaboration of the results. All authors contributed to the article and approved the submitted version.

## Conflict of Interest

The authors declare that the research was conducted in the absence of any commercial or financial relationships that could be construed as a potential conflict of interest.
